# Entropy Extraction from Wearable Sensors for Secure Cryptographic Key Generation in Blockchain and IoT Systems

**DOI:** 10.3390/s25175298

**Published:** 2025-08-26

**Authors:** Miljenko Švarcmajer, Mirko Köhler, Zdravko Krpić, Ivica Lukić

**Affiliations:** Faculty of Electrical Engineering, Computer Science and Information Technology Osijek, Osijek 31000, Croatia; mirko.kohler@ferit.hr (M.K.); zdravko.krpic@ferit.hr (Z.K.); ivica.lukic@ferit.hr (I.L.)

**Keywords:** wearable sensors, smartwatch entropy generation, cryptographic key generation, Samsung Galaxy Watch, blockchain security, physiological and environmental signals, biometric entropy, random number generation

## Abstract

**Highlights:**

**What are the main findings?**

- Entropy generated from smartwatch sensors in shake mode reached Shannon entropy of 0.997 and min-entropy of 0.918, approaching levels of software-based RNGs.
- The proposed method enables fully offline, multisensor entropy harvesting on commercial Wear OS devices using only standard APIs.

**What is the implication of the main finding?**

- Smartwatches can serve as practical, user-controlled entropy sources for local cryptographic key generation, including blockchain transaction signing.
- The approach demonstrates a low-cost, open-source alternative to specialized hardware wallets, aligned with decentralization and self-sovereign identity principles.

**Abstract:**

The increasing demand for decentralized and user-controlled cryptographic key management in blockchain ecosystems has created interest in alternative entropy sources that do not rely on dedicated hardware. This study investigates whether commercial smartwatches can generate sufficient entropy for secure local key generation by utilizing their onboard sensors. An open-source Wear OS application was developed to harvest sensor data in two acquisition modes: still mode, where the device remains stationary, and shake mode, where data collection is triggered by motion events exceeding a predefined acceleration threshold. A total of 4800 still-mode and 4800 shake-mode samples were collected, each producing 11,400 bits of sensor-generated data. Entropy was evaluated using statistical metrics commonly employed in entropy analysis, including Shannon entropy, min-entropy, Markov dependency analysis, and compression-based redundancy estimation. The shake mode achieved Shannon entropy of 0.997 and min-entropy of 0.918, outperforming the still mode (0.991 and 0.851, respectively) and approaching the entropy levels of software-based random number generators. These results demonstrate that smartwatches can act as practical entropy sources for cryptographic applications, provided that appropriate post-processing, such as cryptographic hashing, is applied. The method offers a low-cost, transparent, and user-friendly alternative to specialized hardware wallets, aligning with the principles of decentralization and self-sovereign identity.

## 1. Introduction

Wearable devices, and particularly smartwatches, have become integral to health monitoring, activity recognition, and context-aware computing due to their continuous operation and integration of diverse sensors [1,2]. These sensors generate high-resolution physiological, motion, and environmental data, which, beyond their primary applications, have recently been explored as potential sources of physical entropy in IoT and mobile security contexts [3]. Ensuring high-quality entropy is critical not only for conventional cryptographic applications but also for emerging decentralized systems. Sensor-derived entropy offers an attractive alternative to traditional hardware or software random number generators, as it leverages naturally occurring physical variability that is difficult to replicate or predict externally [4,5].

The secure generation and management of cryptographic keys are foundational to the trust model of blockchain systems [6]. The integrity and authenticity of transactions rely on digital signatures derived from private keys, which must be generated using high-entropy sources to ensure cryptographic strength [7,8]. While traditional methods employ hardware security modules (HSMs), trusted platform modules (TPMs), or cryptographically secure pseudo-random number generators (CSPRNGs), these solutions are often bound to centralized infrastructure, proprietary implementations, or specialized external devices [9,10].

In contrast, users of blockchain-based systems typically favor decentralized, user-controlled, and open-source solutions [11,12]. Privacy, autonomy, and the minimization of trust in third parties are core principles in this domain. Consequently, there is growing interest in leveraging everyday personal devices as secure cryptographic platforms. Among these, the smartwatch presents a compelling opportunity. It is continuously worn, contains diverse motion and environmental sensors, and can operate independently of centralized services [13,14].

The key motivation behind this work lies in bridging the gap between user expectations in blockchain ecosystems and practical cryptographic security. By using entropy derived from physical interactions captured by a smartwatch (e.g., shaking motion), it is hypothesized that sufficient randomness can be harvested to generate private keys suitable for signing blockchain transactions. The approach promotes decentralization, avoids cloud-based key handling, and uses widely available hardware.

To illustrate the alignment between blockchain user values and the proposed approach, a comparison is provided in Table 1.

There is a clear disparity between the expectations of blockchain users and the constraints imposed by traditional key management solutions. Users of decentralized systems consistently demonstrate a preference for local control over cryptographic keys, as reliance on centralized HSMs or cloud-based services introduces risks that conflict with the core principles of decentralization and self-sovereignty [6,11]. The smartwatch-based method addresses this by enabling local key generation and entropy harvesting on the user’s own device.

Privacy and autonomy are strengthened by removing any dependency on external servers. This also eliminates the need for network connectivity during entropy acquisition and key generation. This aligns with the design goals of self-sovereign identity systems, which prioritize the minimization of external trust assumptions. In addition, the use of open-source software remains a critical criterion for adoption in the blockchain community, where transparency and verifiability are highly valued [15]. The proposed method, being platform-accessible and implementable on open devices such as those running Wear OS, supports this requirement.

The physical characteristics of smartwatches—being always-on, body-coupled, and already integrated into daily routines—offer an ergonomic and secure environment for cryptographic operations. Compared to smartphones, smartwatches typically run fewer third-party applications and operate in a more restricted software environment, reducing the potential attack surface and exposure to malicious software. This overcomes the usability and portability limitations of traditional hardware wallets, which often require additional devices and exhibit lower user acceptance [12,16].

Consequently, the smartwatch is positioned as a viable and user-aligned entropy source that addresses both technical requirements and ideological expectations in blockchain ecosystems.

Beyond meeting ideological expectations, the proposed approach derives its technical viability from the unique sensing capabilities of modern smartwatches. These devices include sensors such as accelerometers, gyroscopes, barometers, heart rate monitors, and ambient light sensors. They continuously capture multi-dimensional, time-varying signals. Such signals, particularly when elicited through deliberate user motion (e.g., hand shaking), introduce high levels of physical variability that are difficult to reproduce or predict externally. When properly sampled and conditioned, this sensor data has been shown to contain sufficient entropy to support secure cryptographic key generation [17,18,19].

This reliance on real-world, body-coupled sensor input—rather than computational pseudo-randomness—differentiates the proposed method from conventional approaches, situates it within the broader research agenda of secure entropy harvesting from physical environments. Due to their widespread adoption, ergonomic design, and advanced sensors, smartwatches are a promising but underexplored platform for cryptographic operations in decentralized systems.

The main contributions of this work are as follows:

- Systematic entropy analysis on a commercial smartwatch—to the best of our knowledge, this is the first study to systematically evaluate sensor-derived entropy on a commercial smartwatch in the context of cryptographic key generation for blockchain transaction signing.
- Multisensor entropy harvesting with randomized sensor selection—a novel method is proposed that increases unpredictability by dynamically selecting a subset of sensors in each acquisition session.
- Quantitative evaluation using standardized metrics—entropy is analyzed using statistical measures recommended by NIST SP 800-90B [4], including Shannon entropy, min-entropy, Markov dependency analysis, and compression-based redundancy estimation.
- Comparison with established RNGs—results are compared against hardware- and software-based random number generators, demonstrating competitive entropy levels, particularly in motion-triggered acquisition.
- Practical implementation on a commercially available device—the proposed method is implemented as an open-source Wear OS application running fully offline on a Samsung Galaxy Watch 7 (Samsung Electronics Co., Ltd., Suwon, South Korea), demonstrating that no specialized hardware is required.

The study also has several limitations:

- Data were collected from a single smartwatch model and a single participant, limiting generalizability.
- Only a subset of NIST-recommended tests was applied, with full SP 800-90B validation left for future work.
- Per-sensor entropy contribution was not analyzed in detail, and motion sensors dominate the entropy pool due to their higher sampling rates.

The remainder of this paper is organized as follows. Section 2 reviews related work, providing a categorization of hardware-, software-, and sensor-based entropy generation methods. Section 3 presents the proposed method, describing the system architecture, sensor selection criteria, entropy acquisition protocol, and measurement procedure. Section 4 reports the results of entropy evaluation for still and shake acquisition modes, includes a comparative analysis with established random number generation methods, and discusses the implications of the findings. Finally, Section 5 concludes the study and outlines potential directions for future research.

## 2. Related Work

Reliable cryptographic key generation requires access to high-quality entropy sources that are resistant to prediction, manipulation, and external influence. The generation of such entropy has been the focus of significant research across multiple domains, ranging from low-level hardware implementations to software-based pseudorandom generators and emerging approaches utilizing physiological or environmental data. Each of these methods exhibits distinct trade-offs in terms of security, transparency, hardware dependency, and suitability for mobile or decentralized contexts.

**Figure 1:**
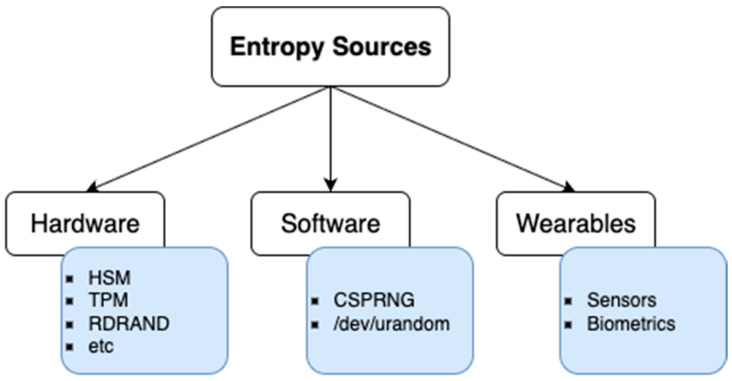
Classification of entropy sources.

In this section, existing approaches to entropy generation are categorized into three main groups as shown in Figure 1. Section 2.1 reviews hardware-based methods, which typically rely on secure elements, TPMs, and hardware wallets equipped with true random number generators (TRNGs). Section 2.2 examines software-based methods, including cryptographically secure pseudorandom number generators (CSPRNGs), deterministic key derivation using mnemonic phrases, and entropy extraction from biometric data. Finally, Section 2.3 focuses on wearable and sensor-based approaches, which exploit human motion, environmental variability, and embedded sensing capabilities to derive entropy directly from physical interactions.

### 2.1. Hardware-Based Entropy Resources

Hardware-based methods for entropy generation rely on physical processes and dedicated components to produce random values with high unpredictability. Such systems are commonly integrated into secure computing environments through elements like hardware security modules (HSMs), trusted platform modules (TPMs), and consumer-grade hardware wallets such as the Ledger Nano or Trezor. These devices typically incorporate TRNGs that operate based on electronic noise, clock jitter, or metastable states in silicon [20].

Ledger hardware wallets, for example, use a certified secure element (e.g., ST33 or ST31 chipsets) that integrates a TRNG in accordance with ISO/IEC 18031 [21] and Common Criteria EAL5+ standards [22,23]. During device initialization, entropy is gathered internally and processed via deterministic key derivation functions to generate BIP39-compliant mnemonic phrases [24,25]. The seed remains confined to the secure element, thereby minimizing attack surfaces from the host system. Similarly, TPMs available in modern computing platforms can generate entropy internally and securely store cryptographic keys for applications such as full disk encryption or TLS [26].

Another example of a hardware-based entropy source is Intel’s RDRAND instruction, available on modern Intel processors. It implements a digital random number generator (DRNG) based on an on-chip TRNG seeding an AES-based deterministic random bit generator (DRBG), following the NIST SP 800-90A standard [9,10]. Although RDRAND provides cryptographically strong randomness in theory, concerns have been raised regarding the transparency and auditability of its internal design, including potential backdoors [7]. As such, its use in security-critical applications is often debated, particularly within communities that prioritize verifiability and open-source principles.

While these approaches offer high entropy quality and resistance to tampering, they present several limitations when viewed from the perspective of decentralization and user autonomy. Most hardware wallets are proprietary, closed-source systems, limiting independent verification of the entropy generation process [12]. Furthermore, they require users to purchase and carry additional devices, which may not align with usability preferences in mobile-first or minimal-hardware contexts. HSMs and TPMs, while suitable for enterprise or server environments, are impractical for personal or wearable deployment due to their physical and infrastructural requirements.

In summary, hardware-based entropy sources are well-established and cryptographically robust, but their dependence on specialized components and limited transparency restricts their suitability for open, user-centric, and mobile cryptographic systems—particularly within decentralized ecosystems such as blockchain.

### 2.2. Software-Based Entropy Generation Methods

Software-based methods for entropy generation and random number derivation are widely employed in cryptographic systems due to their ease of deployment and platform independence. These methods generally rely on deterministic algorithms that are seeded with entropy gathered from various system-level events, user interactions, or hardware noise. Once initialized, such generators produce cryptographically secure pseudorandom numbers (CSPRNGs) that meet statistical criteria for unpredictability and uniformity [10].

One of the most widely adopted software-based approaches is the/dev/urandom interface in Unix-like operating systems, which combines system entropy pools with cryptographic mixing functions to generate pseudorandom output [27]. Although efficient and practical/dev/urandom is not guaranteed to be forward- or backward-secure without sufficient initial entropy, particularly in early boot stages [28]. OpenSSL’s RAND_bytes() and similar functions provide CSPRNG capabilities based on NIST-recommended deterministic random bit generators (DRBGs), but inherit similar limitations regarding their dependence on the quality and availability of initial entropy sources [29].

Stream cipher-based CSPRNGs, such as ChaCha20 as implemented in the libsodium library, have gained popularity due to their performance, simplicity, and resistance to side-channel attacks [30]. These generators are often used for ephemeral key generation, nonce construction, and secure messaging protocols. While their internal state transitions are cryptographically robust, their unpredictability is only as strong as the entropy present in their initial seeding.

In addition to traditional software approaches, biometric and behavioral data—such as keystroke dynamics, heart rate variability, and touchscreen interactions—have been proposed as alternative entropy sources [31]. Although promising, these methods typically require user-specific calibration and may exhibit limited entropy under constrained conditions or in passive collection scenarios.

Overall, software-based methods provide accessible and efficient mechanisms for entropy generation. However, they are fundamentally limited by the quality and trustworthiness of their initial seeding sources, which may themselves be hardware-dependent or opaque. This reinforces the importance of evaluating entropy sources that are both transparent and physically grounded, as explored in wearable sensor-based methods.

### 2.3. Wearable and Sensor-Based Approaches

Recent advances in wearable technology have enabled the integration of a wide range of sensors into compact, body-coupled devices. These sensors, originally intended for health monitoring or activity tracking, have been increasingly explored as entropy sources for cryptographic purposes. The motivation lies in their capacity to capture inherently unpredictable and user-specific physical phenomena, such as hand movements, gait, temperature fluctuations, and cardiovascular signals [32].

Prior research has examined the feasibility of using accelerometer and gyroscope data to generate random sequences by exploiting the stochastic nature of human motion [33]. Other studies have explored entropy extraction from photoplethysmography (PPG) signals, electrodermal activity (EDA), and skin temperature, aiming to leverage biometric variability for key generation [34]. These approaches offer the advantage of continuous, real-time entropy harvesting, which aligns with the operational patterns of modern wearable devices.

Practical deployments remain limited. Many proposed systems rely on custom hardware, controlled environments, or data post-processing steps that hinder real-time applicability. Additionally, some platforms, such as proprietary smartwatches with closed firmware, restrict access to raw sensor data and reduce transparency and reproducibility. Previous studies were primarily focused on biometric authentication, secure pairing, or device identification rather than explicit cryptographic key generation, and to the best of our knowledge, no prior work has systematically evaluated entropy from commercial smartwatch sensors for this purpose.

In contrast, the present work focuses on entropy harvesting from commercially available smartwatches running open platforms (e.g., Wear OS) using native sensors and real-world user interactions. By analyzing motion data in both active (shake) and passive (still) conditions, a quantifiable entropy model is established and compared against traditional entropy sources. This sensor-based method is not only portable and decentralized but also verifiable and open for audit, traits that are particularly valued in blockchain ecosystems.

## 3. Proposed Method

**Figure 2:**
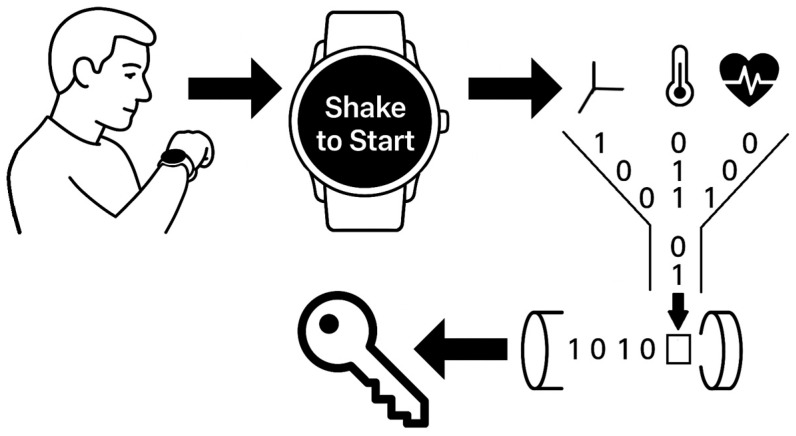
Visual representation of the proposed entropy generation process on a smartwatch.

In this section, the proposed approach for entropy harvesting from smartwatch sensors is presented. The method is designed to enable the local generation of cryptographic entropy on commercially available wearable devices, without the need for external hardware, cloud infrastructure, or platform-specific dependencies. Emphasis is placed on leveraging existing smartwatch onboard sensors to maximize compatibility, portability, and openness of the solution, as shown in Figure 2. The figure illustrates the complete entropy acquisition sequence: the user initiates the process through a shake gesture, multiple onboard sensors capture motion, environmental, and physiological data, the collected signals are vectorized into numerical representations, and these are subsequently processed to produce cryptographic key material.

### 3.1. System Overview

The system operates locally on a commercial smartwatch and collects entropy exclusively from onboard sensors. Two acquisition modes are supported:

- Still mode—data are recorded while the device remains stationary to capture environmental and physiological variability.
- Shake mode—acquisition is triggered by motion events exceeding a predefined acceleration threshold, collecting short bursts of high-frequency data from motion sensors.

In both modes, sensor data are transformed into numerical vectors and processed locally. Each acquisition produces a fixed-size entropy representation and a SHA-256 digest, enabling further analysis and potential cryptographic use. No network connectivity or external hardware is required.

### 3.2. Sensor Selection

The selection of sensors was based on their availability on commercial smartwatches, their capacity to produce non-deterministic data streams, and their relevance to both environmental and physiological variability. Sensors were grouped into three categories according to their origin and signal dynamics:

1. Motion sensors, including the accelerometer, gyroscope, rotation vector, and linear acceleration, capture rapid, user-dependent variations during wrist movement. These sensors exhibit high temporal resolution and significant entropy potential, especially when motion is spontaneous or randomized.
2. Body sensors, such as the heart rate monitor and step counter, provide user-specific physiological signals. While typically more stable than motion data, their variability across time and individuals can still contribute valuable entropy, particularly in multimodal fusion contexts.
3. Environmental sensors, such as the barometric pressure sensor, measure external physical conditions. These signals vary subtly over time and can introduce low-frequency noise that increases unpredictability in passive (still) acquisition scenarios.

In this study, a total of eight sensors were selected based on their prevalence across modern smartwatches (especially those running Wear OS), their accessibility via standard APIs, and a preliminary review of device hardware specifications from manufacturers such as Samsung and Google. The selected sensors were as follows:

1. Accelerometer
2. Gyroscope
3. Rotation Vector Sensor
4. Linear Acceleration Sensor
5. Gravity Sensor
6. Barometer (Pressure Sensor)
7. Heart Rate Sensor
8. Step Counter

These sensors were chosen due to their ubiquity, dynamic behavior, and low power requirements. According to the official Android developer documentation and public specifications of devices such as the Samsung Galaxy Watch 5/6/7 and Google Pixel Watch 2 (Google LLC, Mountain View, CA, USA), these are consistently available and produce sufficient signal entropy under both still and active conditions [35,36]. An overview of their typical sampling frequencies and the expected number of measurements within a single 2.2 s acquisition window is provided in Table 2.

All onboard sensors are factory-calibrated according to Samsung manufacturer specifications [35], ensuring baseline measurement accuracy without the need for user-performed calibration.

The decision to utilize a randomized subset of active sensors during each session was made to increase entropy diversity and to mitigate predictability. This dynamic selection further prevents adversaries from replicating sensor access patterns or tailoring attacks to fixed data sources.

All selected sensors are accessible via the Android Wear OS SensorManager API without requiring elevated permissions, ensuring user-level deployment feasibility. Access to these sensors was managed entirely through the standard Android/Wear OS permission framework. At runtime, the system prompts the user to explicitly grant the necessary permissions (e.g., BODY_SENSORS for heart rate, ACTIVITY_RECOGNITION for motion data, and general sensor access for inertial and environmental sensors). These permissions are requested through standard Android dialog boxes, and data collection cannot proceed without user approval. Once granted, the application registers listeners through the Android SensorManager API, which delivers real-time data streams only from the sensors authorized by the user. This guarantees that all acquisitions remain subject to explicit consent, enforced at the OS level, and that no restricted or undocumented system functions are bypassed. Importantly, the combination of diverse sensor types enhances entropy quality through data fusion, as statistical correlations between sensor modalities are minimal, especially during short sampling windows.

**Figure 3:**
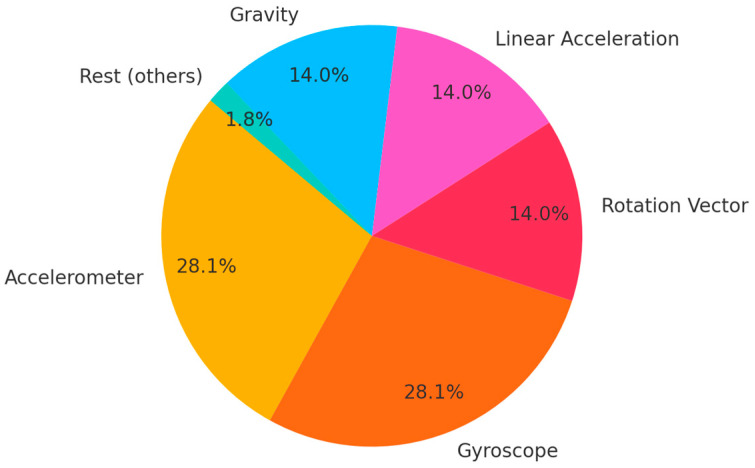
Expected percentage contribution of each sensor type to the total data volume collected during a 2.2 s acquisition window, based on typical sampling frequencies listed in Table 2.

The relative contribution of each sensor to the total amount of data collected in a single 2.2 s acquisition window is shown in Figure 3. Motion sensors such as the accelerometer and gyroscope dominate the data volume due to their high sampling rates, whereas environmental and physiological sensors (barometer, heart rate, and step counter) contribute significantly less.

Despite their lower sampling rates, the inclusion of these sensors is recommended because they provide heterogeneous, physically independent signal sources. This diversity increases overall entropy by introducing additional stochastic variability and reducing the risk of correlated patterns across sensor modalities.

### 3.3. Entropy Acquisition Protocol

The entropy acquisition process is governed by a lightweight protocol implemented entirely on the smartwatch, designed to operate autonomously and without network connectivity. Two distinct acquisition modes were defined to capture different entropy profiles:

1. Still mode: Sensor data are collected over a fixed time window starting immediately upon application launch, while the user remains stationary. This mode targets slow-changing environmental and physiological inputs such as pressure, heart rate, and gravity.
2. Shake mode: Acquisition is event-driven and triggered by a motion-detection threshold. In this study, the threshold for acceleration magnitude was empirically set to 8.0 m/s^2^ based on preliminary experiments to balance sensitivity and noise rejection.
  This value ensured reliable detection of intentional shake gestures while minimizing false triggers from normal wrist movements.
  Upon detecting a change in acceleration magnitude, a short burst of high-frequency data is collected from selected motion sensors.

Each acquisition session begins with the randomized selection of a subset of sensors from the list defined in Section 3.2. This randomization introduces additional unpredictability and hinders potential adversaries from anticipating sensor combinations.

**Figure 4:**
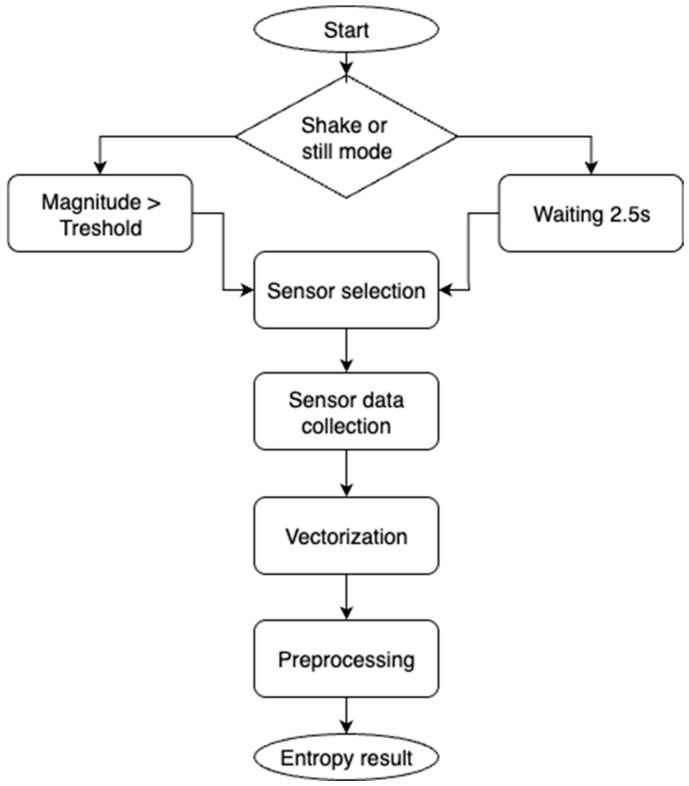
Flowchart of the proposed entropy acquisition pipeline for both shake and still modes.

Once triggered, the application performs the following steps, shown in Figure 4:

1. Sampling: Raw sensor data are collected using the Android SensorManager API, with typical sampling rates ranging from 50 Hz to 200 Hz, depending on sensor type. The acquisition window was set to 2.2 s to ensure that each selected sensor provides at least one complete measurement cycle, as determined from the manufacturer’s specifications for update frequencies. An additional 0.3-s pause between sessions was introduced to ensure that sensor buffers were flushed before the next acquisition.
2. Vectorization: Each data frame is transformed into a structured 1D numerical vector and concatenated with a timestamp and sensor identifier. This produces a uniform and reproducible representation of heterogeneous sensor streams1.
3. Preprocessing: Basic normalization is applied to account for sensor scaling, and values are discretized to 8-bit resolution to align with common entropy evaluation practices (e.g., NIST SP 800-90B), reducing high-frequency sensor noise and enabling direct applicability of standardized statistical tests.
4. Hashing: The final vectorized output is processed through a cryptographic hash function (SHA-256) to produce a fixed-length digest, which is treated as the entropy-bearing seed or candidate cryptographic key.

All steps are performed entirely offline, and no data leaves the device at any point. Logging is used internally to record entropy metrics for evaluation purposes but remains sandboxed within the application environment. This approach ensures privacy, replicability, and full user control over the entropy generation process.

### 3.4. Implementation Details

The proposed entropy acquisition system was implemented as a standalone application for smartwatches running Wear OS. The application was developed using Kotlin (version 1.9.23) and Jetpack Compose (version 1.7.0), following modern Android development practices and targeting API level 30 and above. This ensured a responsive and efficient user interface suitable for low-power wearable environments.

The application’s lifecycle is minimal and fully event-driven. Upon first launch, the app detects all available onboard sensors using the SensorManager API and performs a one-time random shuffle to define the acquisition sequence. This randomized order remains fixed throughout each session to ensure consistency in entropy sampling.

For user interaction, a minimal interface is presented, displaying the message “Shake to Start” once initialization is complete. When the device detects a motion exceeding a predefined acceleration threshold, a short data acquisition window is triggered. During this window, data are collected asynchronously from the selected subset of sensors.

Each sampling session produces a single entry stored in a CSV file within the application sandbox. This entry includes the following:

1. A sequence index,
2. A transformed numerical representation of the collected sensor data (as a single entropy vector or derived scalar),
3. Timestamp (optional).

The application operates fully locally on the smartwatch, ensuring user privacy and full control over the collected data.

For entropy assessment and cryptographic relevance, each collected sensor vector is hashed using the SHA-256 algorithm, generating a fixed-length digest suitable for seeding key material. These digests are used exclusively for evaluation and remain confined to local storage.

The application was evaluated on a Samsung Galaxy Watch 7, which provided full compatibility with the Wear OS platform and offered unrestricted access to all required sensor interfaces. This smartwatch was chosen as the test device due to its exceptional suitability for entropy-related experimentation: it supports open app deployment, features a wide array of onboard sensors, and is well-documented within the Android development ecosystem.

From a security perspective, the potential attack surface is inherently reduced since all entropy harvesting, processing, and key generation steps are executed entirely on the smartwatch. No raw sensor data or intermediate entropy values are transmitted to external devices or networks, which significantly limits opportunities for interception or manipulation during the acquisition process.

The full source code is publicly available on GitHub, enabling independent verification of the data acquisition process and reproducibility of the experiments without exposing sensitive raw sensor data.

**Figure 5:**
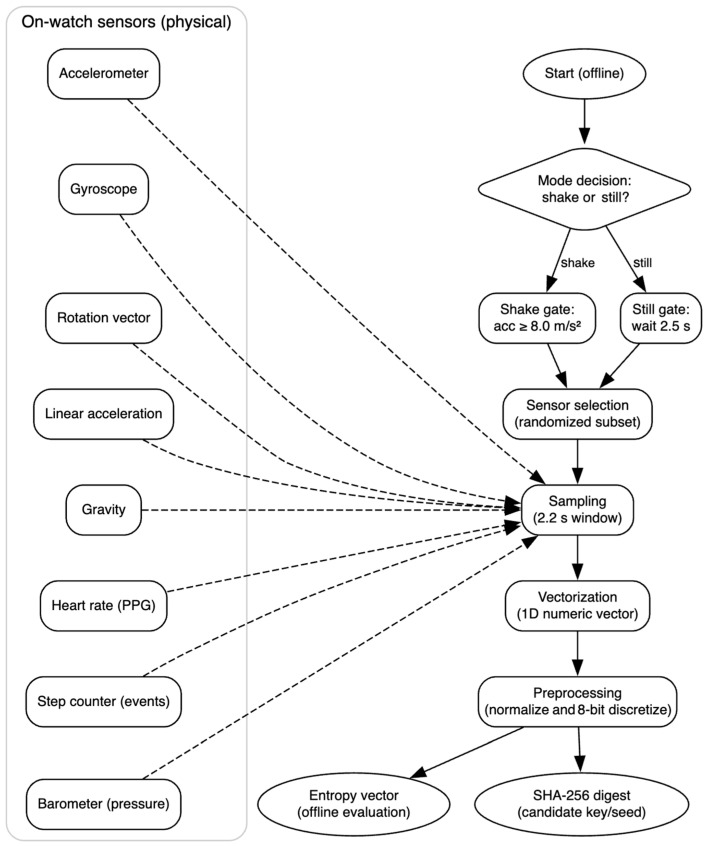
System architecture for entropy acquisition and processing on a commercial smartwatch.

Figure 5 provides an overview of the proposed system architecture, showing the interactions between on-watch physical sensors, acquisition mode logic, and processing modules. This complements the procedural details described in Section 3.3 and Section 3.4.

### 3.5. Measurement Procedure

The measurement procedure was conducted in two phases: an initial phase aimed at application refinement and a final phase dedicated to systematic entropy evaluation.

#### 3.5.1. Preliminary Testing

Several iterations of the application were tested to refine sensor handling, data transformation, and motion detection logic. Approximately 1000 preliminary samples were collected during this phase. These were used exclusively for debugging and early entropy estimation and were excluded from the final statistical analysis.

#### 3.5.2. Final Measurements—Still Mode

The first final measurement phase targeted the still mode, with the smartwatch placed motionless on a desk to minimize user-induced variability. Entropy in this mode was therefore primarily derived from sensor noise and subtle environmental micro-variations.

- A total of 5000 measurements were initially recorded.
- Each measurement lasted 2.2 s and produced 11,400 bits of sensor-derived data.
- Successive measurements were automatically triggered with a 0.3-s delay.
- Before the completion of the 5000 still-mode measurements, the smartwatch was inadvertently displaced on the desk. To ensure consistency, the last 200 measurements were excluded from the analysis, leaving 4800 valid samples, which was more than sufficient for statistical testing.

#### 3.5.3. Final Measurements—Shake Mode

In the second phase, the smartwatch was worn on the wrist of a single participant performing daily activities. Data acquisition was event-driven and triggered only when wrist motion exceeded a predefined acceleration threshold (as described in Section 3.3). Each trigger produced one entropy sample under the same sampling and formatting conditions as in still mode. A total of 4800 samples were collected in this mode.

**Figure 6:**
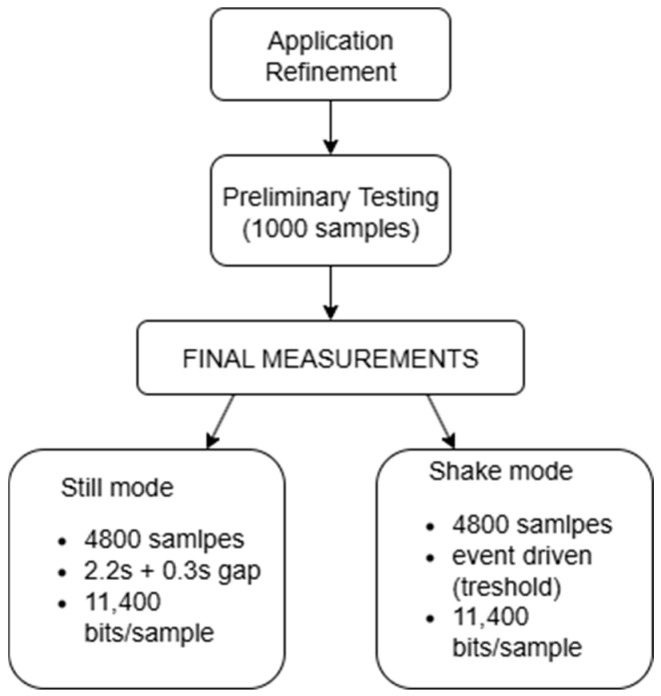
Experimental workflow for still and shake entropy acquisition.

All measurements were stored in CSV format, with one line per sample containing the sequence index, the numerical entropy vector, and its locally computed SHA-256 digest. No preprocessing was applied beyond normalization and discretization (Section 3.3), ensuring that entropy analysis reflected raw sensor variability. Datasets were exported manually for offline statistical evaluation. The experimental workflow is summarized in Figure 6.

## 4. Analysis of Results

This section presents the entropy analysis of the proposed smartwatch-based method for still and shake acquisition modes. Several statistical metrics commonly used in entropy evaluation, including measures recommended by NIST SP 800-90B, were applied to assess the unpredictability of the collected data.

The obtained results are compared with established hardware- and software-based entropy sources to evaluate the cryptographic suitability of the method.

### 4.1. Entropy Evaluation Methodology

The statistical evaluation of the smartwatch sensor data was conducted in accordance with widely accepted practices for assessing entropy sources, with reference to the guidelines outlined in NIST SP 800-90B [4]. Since the goal of the study was to determine whether sensor-derived data could provide sufficient unpredictability for cryptographic key generation, multiple complementary metrics were employed to capture different aspects of randomness and statistical independence.

Four primary evaluation methods were applied:

1. **Shannon Entropy**
  Shannon entropy was computed according to (1) to quantify the average information content per symbol in the collected sequences. This metric is widely used as an indicator of randomness quality, as it reflects how uniformly the observed symbols are distributed [37]. Although NIST SP 800-90B does not recommend it as a primary security metric due to its sensitivity to distributional assumptions, it provides a valuable initial assessment of entropy sources.
  (1) $$H_{\mathrm{Shannon}}=-\sum_{i=1}^{n}p_{i}\log_{2}(\mathrm{pi})$$
  Here, *p_i_* represents the empirical probability of symbol *i*, and *n* is the total number of distinct symbols. Higher entropy values indicate a more uniform distribution and therefore greater unpredictability.
2. **Min-Entropy**
  Min-entropy was calculated according to (2) to estimate the worst-case unpredictability of the sensor-derived sequences. Unlike Shannon entropy, which measures the average information content, min-entropy focuses on the most probable symbol and therefore provides a conservative lower bound on the true entropy of a source. This makes it particularly relevant for cryptographic applications, as recommended by NIST SP 800-90B [38].
  (2) $$H_{\min}=-\log_{2}(\max_{i}P_{i})$$
  Here, max_i_P_i_ is the highest empirical probability observed among all symbols. Lower maximum probabilities correspond to higher min-entropy values, indicating reduced predictability and greater suitability for secure key generation.
3. **Markov Dependency Analysis**
  To account for potential temporal correlations between successive samples, a first-order Markov model was used to estimate the transition probabilities between states [39]. This method is recommended for non-IID (non-independent and identically distributed) sources, which are typical for physical sensor measurements.
4. **Compression-Based Redundancy Estimation (GZIP Test)**
  Compression-based methods are often used as heuristic indicators of structural regularities in data [40]. In this study, a standard GZIP algorithm based on LZ77 compression was applied; sequences with higher entropy are expected to show lower compression ratios. While not formally equivalent to the compression test described in NIST SP 800-90B, the underlying principle is comparable [41].

The selected metrics were chosen because they capture both global distributional properties (Shannon, GZIP) and conservative worst-case guarantees (min-entropy, Markov). More advanced NIST-defined tests, such as the most common value (MCV) test or restart/adaptive proportion tests, were not applied in this initial study and are planned for future work.

All metrics were computed on raw sensor-derived vectors exported directly from the smartwatch, without post-processing beyond the basic normalization described in Section 3.3.

### 4.2. Results—Smartwatch Entropy (Still vs. Shake)

The entropy of the collected data was evaluated separately for still and shake modes. Four statistical metrics were applied: Shannon entropy, min-entropy, Markov dependency analysis, and GZIP compression ratio. These metrics provide complementary perspectives on randomness, capturing both average information content and worst-case predictability. The results are summarized in Table 3.

**Figure 7:**
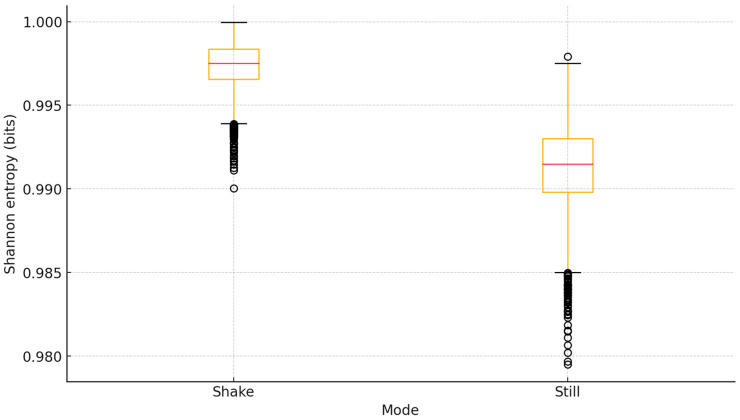
Boxplot comparison of Shannon entropy values for shake and still acquisition modes.

The shake mode consistently outperformed the still mode across all metrics, reflecting the higher variability and reduced temporal correlation introduced by spontaneous wrist motion. Figure 7 shows the distribution of Shannon entropy values (computed according to (1)) for both modes, with the shake mode exhibiting a slightly higher median and a narrower interquartile range. This indicates that motion-triggered acquisition not only improves the average information content but also provides more stable randomness quality across individual samples.

**Figure 8:**
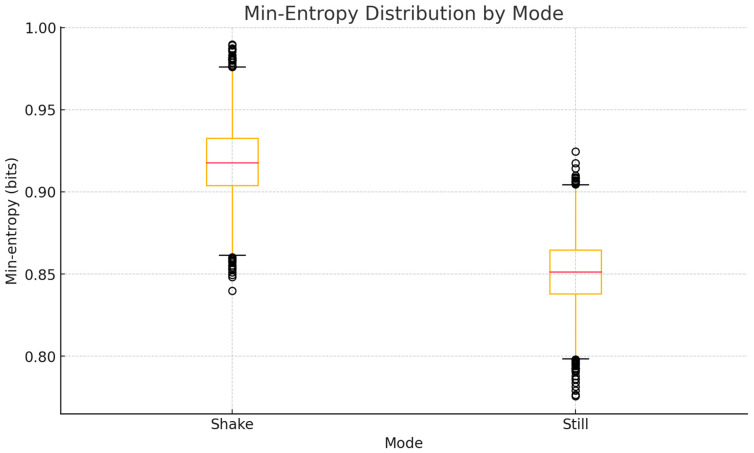
Boxplot comparison of min-entropy values for shake and still acquisition modes.

The min-entropy distribution (Figure 8) reveals a more pronounced difference between the two modes. Still-mode samples show a wider spread and a lower median value, indicating stronger predictability and the presence of repeated patterns. In contrast, shake-mode samples cluster around higher values, demonstrating improved worst-case unpredictability, which is critical for cryptographic applications where the weakest entropy segment determines overall security.

Notably, the min-entropy of 0.918 (computed according to (2)) approaches levels typically observed in hardware-based random sources, indicating potential suitability for cryptographic applications. The lower GZIP compression ratio in still mode further confirms the presence of more regular patterns when the device is stationary.

These findings suggest that motion-driven acquisition provides significantly stronger entropy, while still-mode data may serve as a supplementary or fallback source under passive conditions.

### 4.3. Comparative Analysis with Other Methods

To assess the cryptographic relevance of the smartwatch-based entropy source, the obtained results were compared with representative hardware- and software-based random number generators. The comparison includes TRNGs, CSPRNGs, and commonly used operating system RNGs. Metrics for these established methods were taken from publicly available evaluations and previous studies and compared in Table 4.

The shake mode results are competitive with operating system RNGs, showing only a minor deficit in min-entropy compared to/dev/urandom. While still below TRNG or CSPRNG levels, the smartwatch-based method demonstrates strong unpredictability given its reliance solely on locally harvested physical signals.

The still mode, although weaker, remains within acceptable entropy levels for non-critical randomness applications. Both modes benefit from decentralization, transparency, and the absence of external dependencies, which are key requirements for privacy-sensitive blockchain ecosystems.

These results demonstrate that a commodity smartwatch, without any dedicated cryptographic hardware, can deliver entropy levels approaching those of widely trusted OS RNGs and even nearing the lower range of TRNG performance. This finding is significant because it proves that secure, high-quality randomness can be sourced from devices that many blockchain users already own, thereby reducing cost barriers and removing the need for specialized external modules. The implications extend to decentralized systems and privacy-sensitive environments, where local and verifiable entropy generation directly on the user’s device mitigates supply-chain trust issues and centralization risks inherent in proprietary RNGs.

## 5. Discussion

The results indicate that smartwatch sensors, particularly in motion-triggered (shake) mode, can serve as a viable source of cryptographic entropy. The observed Shannon entropy (0.997) and min-entropy (0.918) are comparable to software-based random number generators such as/dev/urandom and approach the lower bound of hardware-based TRNGs. This level of unpredictability is considered sufficient for many cryptographic operations, including key generation for blockchain transaction signing, provided that additional post-processing (e.g., cryptographic hashing or key-stretching) is applied [1,2].

The still mode produced weaker results, with a noticeably lower min-entropy (0.851) and higher data regularity, as confirmed by the compression analysis. Nevertheless, even this mode can supplement entropy pools in scenarios where active user interaction is not possible. Its inclusion reflects the practical need for passive entropy harvesting in wearables.

The Markov analysis confirmed that shake-mode data exhibit lower temporal correlations than still-mode data, validating the assumption that user-driven motion introduces additional randomness. This is particularly relevant for non-IID entropy sources, which are common in physical systems [3].

Although human body motion or physiological indicators may seem externally observable, the entropy acquisition process is based on short 2.2-s sampling windows combined with randomized sensor subset selection. Within such a narrow time frame, it is practically infeasible to reproduce identical micro-variations in wrist acceleration, rotational dynamics, barometric pressure changes, and heart rate fluctuations. The unpredictability is further amplified by the fact that the active set of sensors differs across acquisition sessions and that their outputs are concatenated into entropy vectors according to the order in which measurements are received by the system. This results in subtle but irreproducible variations in vector structure. Consequently, even if gross user movements could be monitored from a distance, it would remain extremely difficult to replicate the exact multidimensional and temporally ordered conditions necessary to obtain the same entropy output.

Although the smartwatch-based method does not yet match the theoretical security of dedicated TRNGs or mature CSPRNGs, it offers unique advantages aligned with blockchain user requirements:

- Decentralization and local control—entropy is generated and used entirely on the user’s own device, without reliance on centralized hardware or servers.
- Transparency and auditability—open-source implementation allows public verification, unlike closed systems such as Intel RDRAND.
- Practicality and accessibility—the method leverages existing consumer hardware, removing the need for specialized wallets or external RNG modules.

Future work will include a full NIST SP 800-90B evaluation, extended multi-user testing, and optimization of sensor fusion techniques to further improve min-entropy. These steps are expected to establish the method as a credible alternative for lightweight, user-centric cryptographic applications.

## 6. Conclusions

This study demonstrated that commercially available smartwatches can serve as practical entropy sources for cryptographic applications, including blockchain transaction signing. By harvesting data from multiple onboard sensors, two acquisition modes—still and shake—were evaluated. The shake mode achieved Shannon entropy of 0.997 and min-entropy of 0.918, values comparable to established software-based random number generators, while the still mode, although weaker, provided a consistent supplementary entropy source.

The method offers several advantages relevant to blockchain ecosystems: it operates fully offline, requires no specialized hardware, and supports open-source implementation, aligning with the principles of decentralization and self-sovereign identity. Although the entropy levels do not yet reach those of dedicated TRNGs, the results indicate that, with appropriate post-processing (e.g., cryptographic hashing), smartwatch-generated entropy is sufficient for secure local key generation.

Several limitations should be acknowledged. The current evaluation was conducted using a single smartwatch model and participant, restricting the generalizability of the results. Only a subset of NIST SP 800-90B tests was applied, and no direct comparison with medical-grade measurement devices was performed, although the smartwatch sensors are factory-calibrated according to manufacturer specifications. A detailed security analysis was not conducted; however, the potential attack surface is significantly reduced by the fact that entropy harvesting and key generation occur entirely on the smartwatch without transmitting raw data externally.

Future work will expand testing to multi-user datasets, analyze entropy contributions of individual sensors, apply the complete NIST SP 800-90B framework, and include adversarial testing to evaluate robustness against targeted attacks. Additionally, a comparative analysis of power consumption and computational efficiency will be conducted to assess the practicality of long-term use. These steps aim to establish smartwatch-based entropy generation as a credible, user-friendly, and energy-efficient alternative to traditional key management solutions in security-critical domains.

## Figures and Tables

**Table 1:** Comparison of blockchain user preferences with traditional approaches and the proposed smartwatch-based method.

User Preferences	Traditional Approach	Proposed (Smartwatch)
Decentralized key control	HSM/Cloud dependent	Local on device
Privacy/no central entity	May rely on servers	No network dependency
Open-source compatibility	Often proprietary	Fully open implementation
No extra hardware	Requires special wallet	Uses existing smartwatch
Always-on/mobile	Desktop or specialized hardware required	Wrist-worn, always present

**Table 2:** Typical sampling frequencies and expected number of measurements per 2.2 s acquisition window for each selected Galaxy watch 7 sensors.

Sensor	Typical Frequency (Hz)	Expected Samples
Accelerometer	100	220
Gyroscope	100	220
Rotation Vector	50	110
Linear Acceleration	50	110
Gravity	50	110
Barometer (Pressure)	5	11
Heart Rate	1	2
Step Counter	Event-driven (~0.5 Hz)	~1

**Table 3:** Comparative entropy evaluation metrics for still and shake acquisition modes.

Metric	Still Mode	Shake Mode
Shannon entropy (H)	0.991	0.997
Min-entropy (Hmin)	0.851	0.918
Markov dependency (avg. state prob.)	Moderate correlation	Low correlation
GZIP compression ratio	0.72	0.95

**Table 4:** Comparative evaluation of the proposed smartwatch-based entropy generation approach against established entropy sources.

Method/Source	Shannon Entropy	Min-Entropy	Notes
TRNG (e.g., Intel RDRAND)	≈0.999	≈0.998	Hardware, closed-source [9]
CSPRNG (ChaCha20)	≈0.999	≈0.997	Software, deterministic but cryptographically secure [30]
OS RNG (/dev/urandom)	≈0.995	≈0.950	Mixed hardware/software seeding [28]
Smartwatch—Still mode	0.991	0.851	Passive, environmental, and body sensors (this work)
Smartwatch—Shake mode	0.997	0.918	Motion-triggered, multisensor fusion (this work)

## Data Availability

The data and source code supporting the findings of this study are publicly available at https://github.com/msvarcmajer/Entropy_samsung (accessed on 25 August 2025). The repository contains all sensor datasets in CSV format and the Wear OS application source code used for data acquisition and entropy evaluation.
